# Supplementary material for: Cryo-EM structure of the human ferritin–transferrin receptor 1 complex
Source: Nat Commun. 2019 Mar 8;10:1121. doi: 10.1038/s41467-019-09098-w (PMC6408514; doi:10.1038/s41467-019-09098-w)
Supplement: Supplementary file 1 — Supplementary Information [file 41467_2019_9098_MOESM1_ESM.pdf]

Supplementary Materials for

Cryo-EM structure of the human Ferritin-Transferrin Receptor 1 complex

L.C. Montemiglio, C. Testi, et al.

This PDF file includes:

Supplementary Figure 1 to 6

Supplementary Table 1 to 6

Supplementary References

Supplementary Information

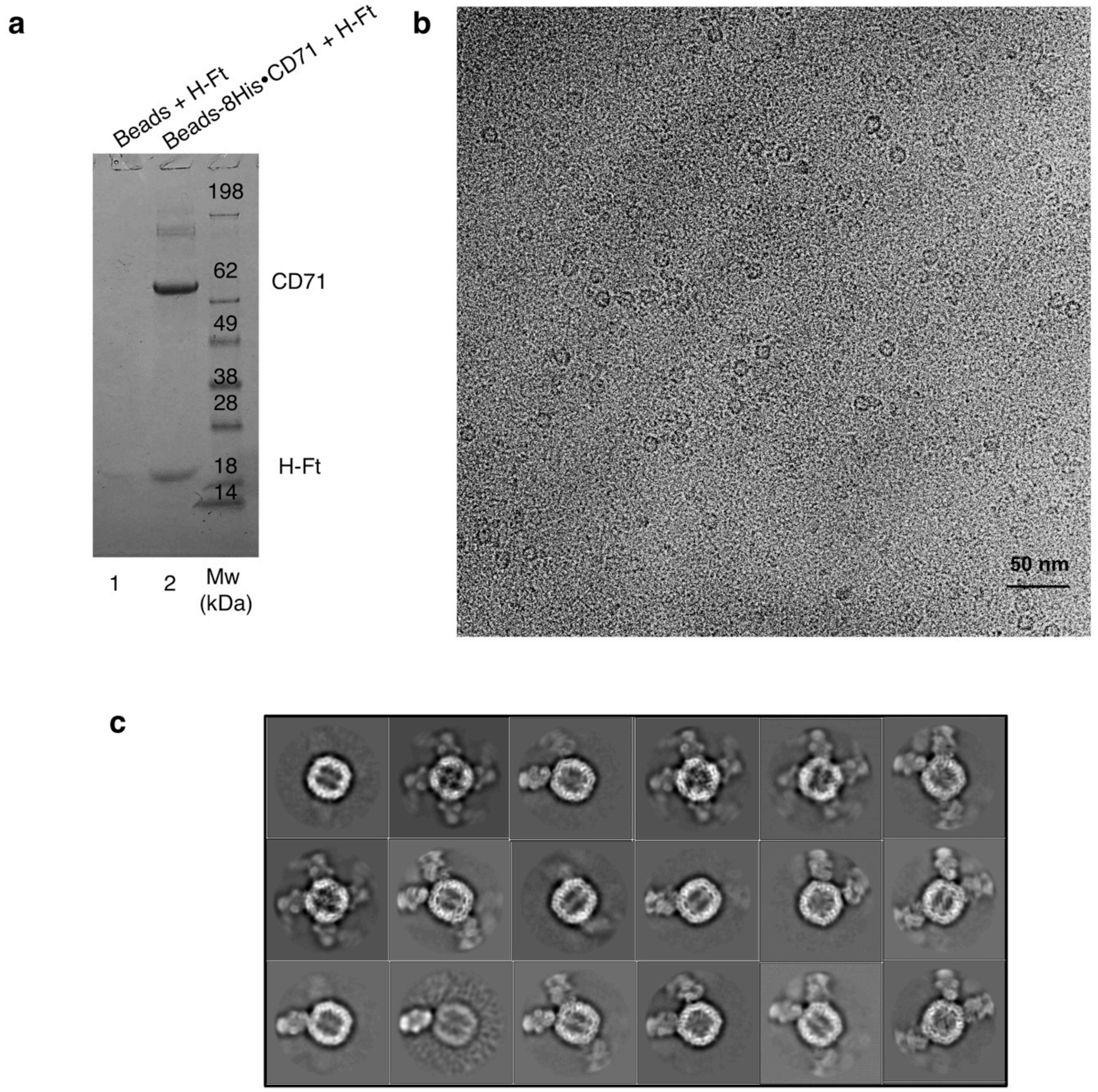

Supplementary Figure 1. CD71/H-Ft complex preparation, cryo-EM images and 2D averages. a. Pull-down assay: CD71/H-Ft complex preparation. SDS-PAGE of proteins eluted with 290 mM imidazole from TALON resin beads not treated (lane 1) and treated (lane 2) with His-tagged CD71 (8His·CD71, monomer Mw~75 kDa) followed by H-Ft (monomer Mw~21 kDa). H-Ft shows negligible level of non-specific binding to the beads in the absence of CD71. H-Ft binding results markedly enhanced when CD71 is bound to the resin. Gel was stained by Coomassie blue and it is shown in greyscale color. Molecular Weight (Mw) Marker: SeeBlue Prestained Standard (Thermo Fisher Scientific, USA). Source data are provided as a Source Data file. **b.** Representative micrograph of CD71/H-Ft. **c.** Representative 2D class averages as calculated with RELION.

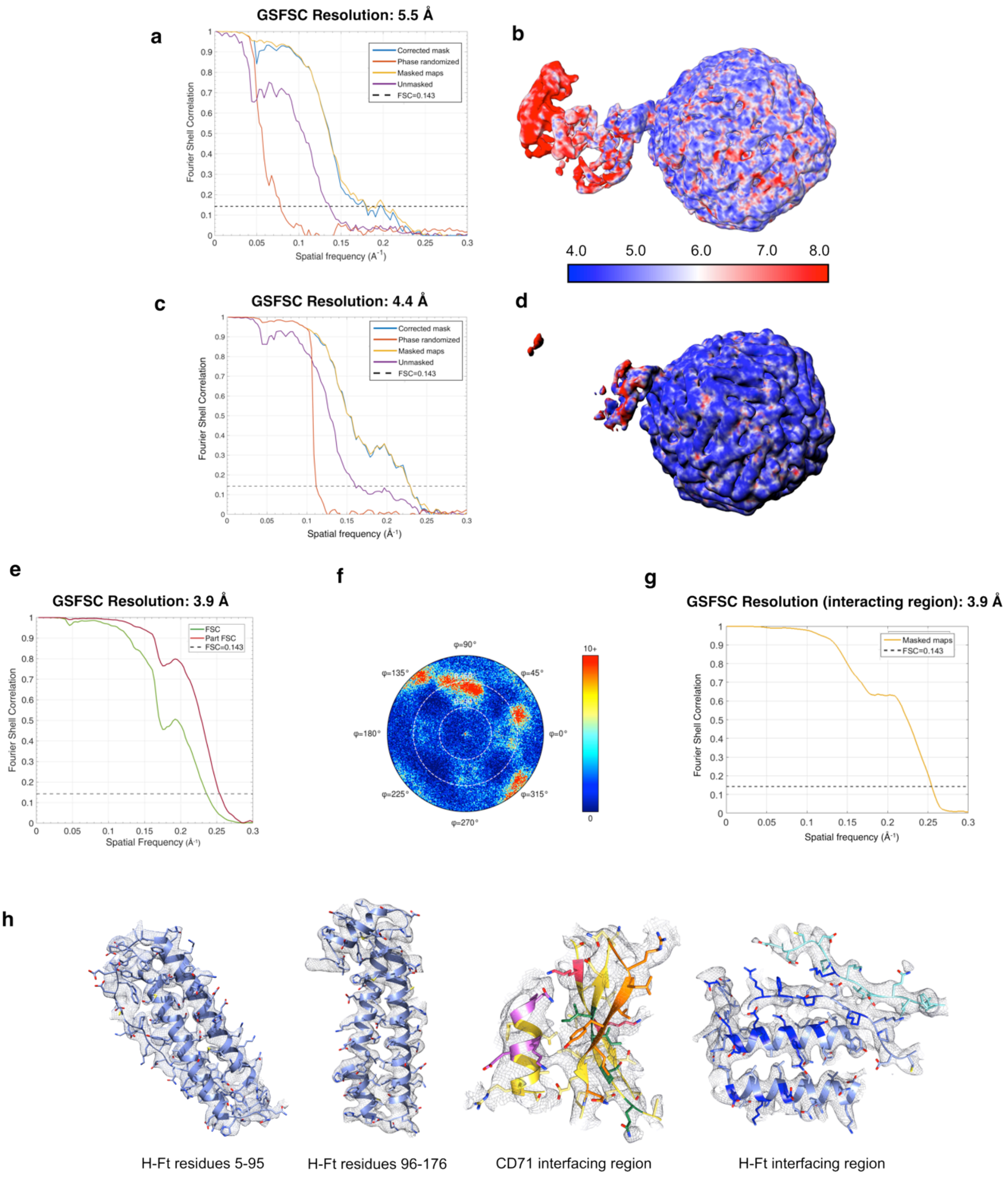

Supplementary Figure 2. CD71/H-Ft complex cryo-EM resolution map. a, c. Gold standard FSC curves of the global maps at 5.5 Å (**a**) and 4.4 Å (**c**) calculated in RELION. In the 5.5 Å map, both CD71 and H-Ft were visible at the level of their secondary structures. **b, d.** The final 5.5 (**b**) and 4.4 Å (**c**) maps are colored according to local resolution estimated by RESMAP. **e, f.** Gold standard FSC curves of the final global map at 3.9 Å (**e**) and angular distribution of particle projections calculated in cisTEM (**f**). Color bar: red represents the most populated projection (+10 particles) and blue the least populated. **g.** FSC curve of the electron density map which only includes the contact region between CD71 ectodomain and H-Ft. The resolution is 3.9 Å based on the gold-standard 0.143 FSC criterion. **h.** Representative cryo-EM density of different parts of H-Ft and for the interfacing regions of CD71 and H-Ft.

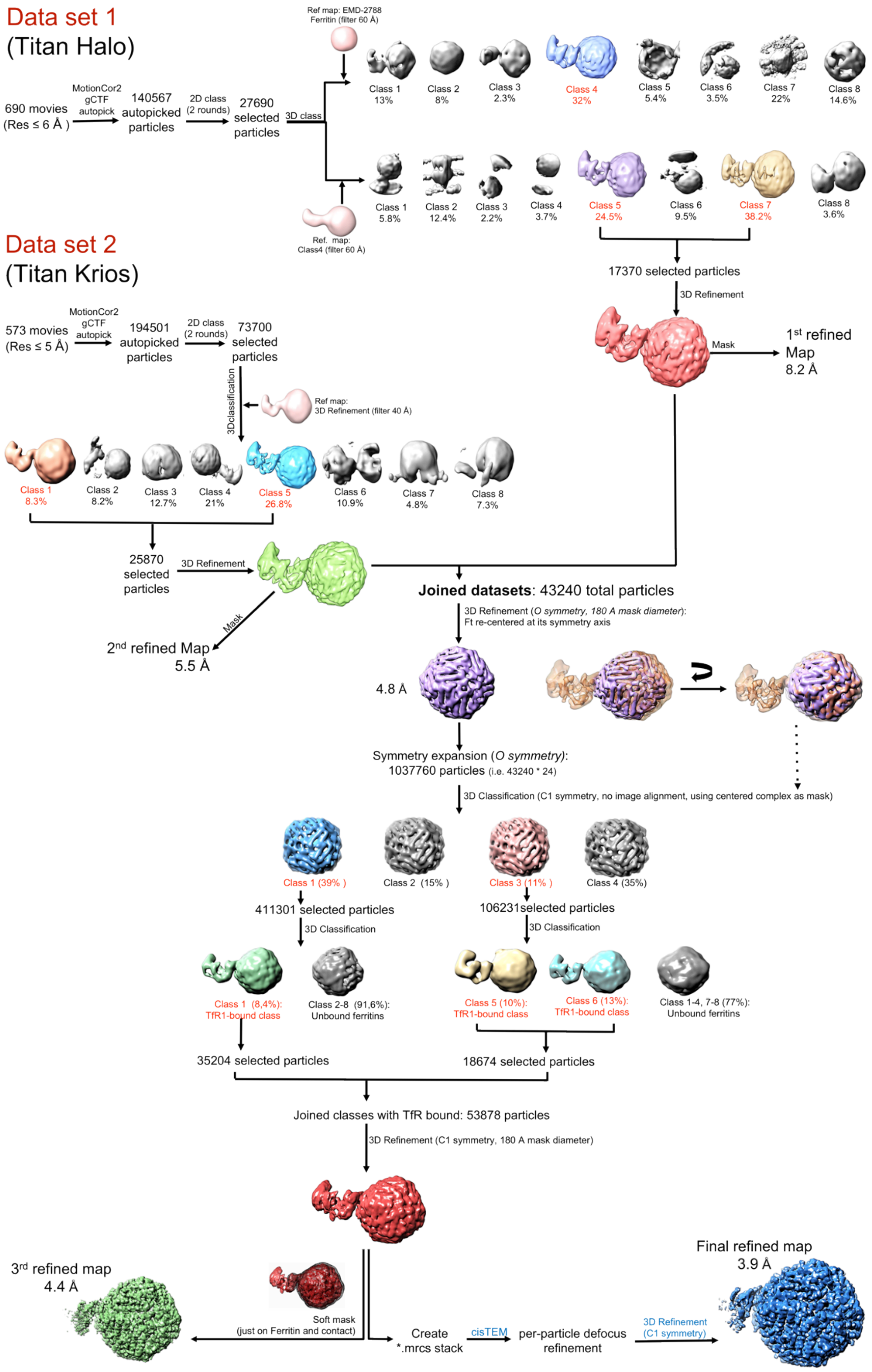

Supplementary Figure 3. Data processing workflow for CD71/H-Ft complex. Schematic diagram of dataset 1 and 2 processing for the 5.5 Å, 4.4 Å global map (obtained with RELION) and the 3.9 Å final map (obtained with cisTEM). Volume density is displayed for each class and final refinement as well as percentage distribution and number of particles.

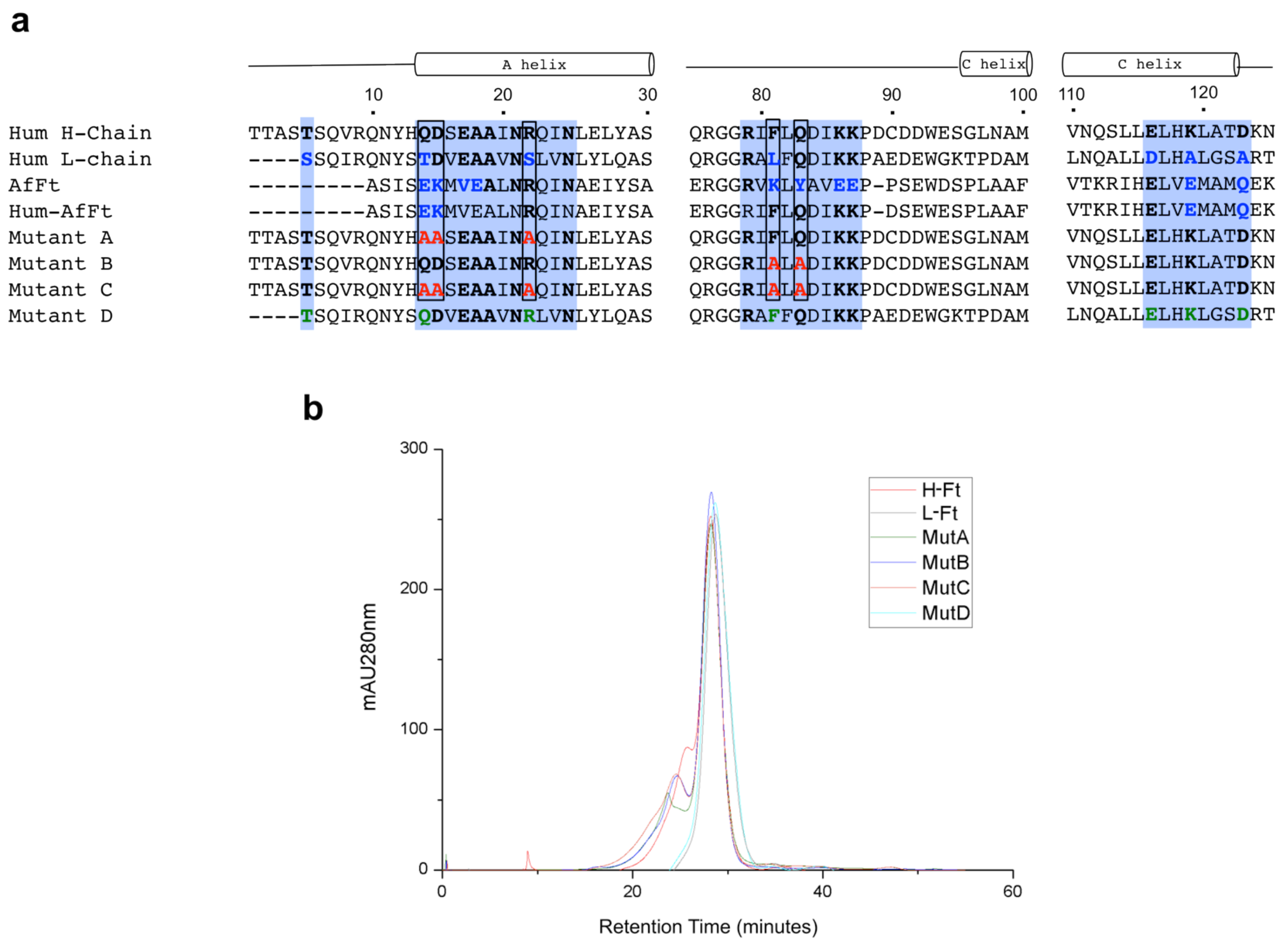

Supplementary Figure 4. Structure-based sequence alignment of ferritins and 24-mer assembly assessment. a. Partial structure-based sequence alignment of the regions contacting CD71 receptor of human H- (Hum H-chain) and L-chain (Hum L-chain) ferritins, *A. fulgidus* (AfFt) and humanized *A. fulgidus* (Hum-AfFt) ferritins, mutant A, B, C and D (amino acids 1-30, 70-100 and 110-125) was made using ClustalW2 [1]. Elements of secondary structure of human H-Ft are shown on the top, using cylinder representation for α-helices. Colored background highlights the H-Ft contact regions with CD71; black bold residues indicate interacting human H-Ft amino acids and conserved among ferritins; for other ferritins, not conserved residues are shown in blue and mutated residues are in red. Mutations introduced to convert the Hum L-chain in Hum H-chain are in green (Mutant D). Black boxes indicate residues identified as critical for the CD71 epitope recognition. **b.** Gel filtration chromatographic profile of ferritins utilized in this study. A main peak at retention times typical of the human ferritin wild type (28.2 min) is present, thus proving that all mutants retain the 24-mer assembly of the human wild type ferritins. In addition, a pre-peak at about 25 min is also observed in the H-chain constructs. This was previously ascribed to the presence of cysteine residues (2 per subunit, 48 for 24-mer) on the surface of the H-chain ferritins that are absent in the L-chain proteins [2].

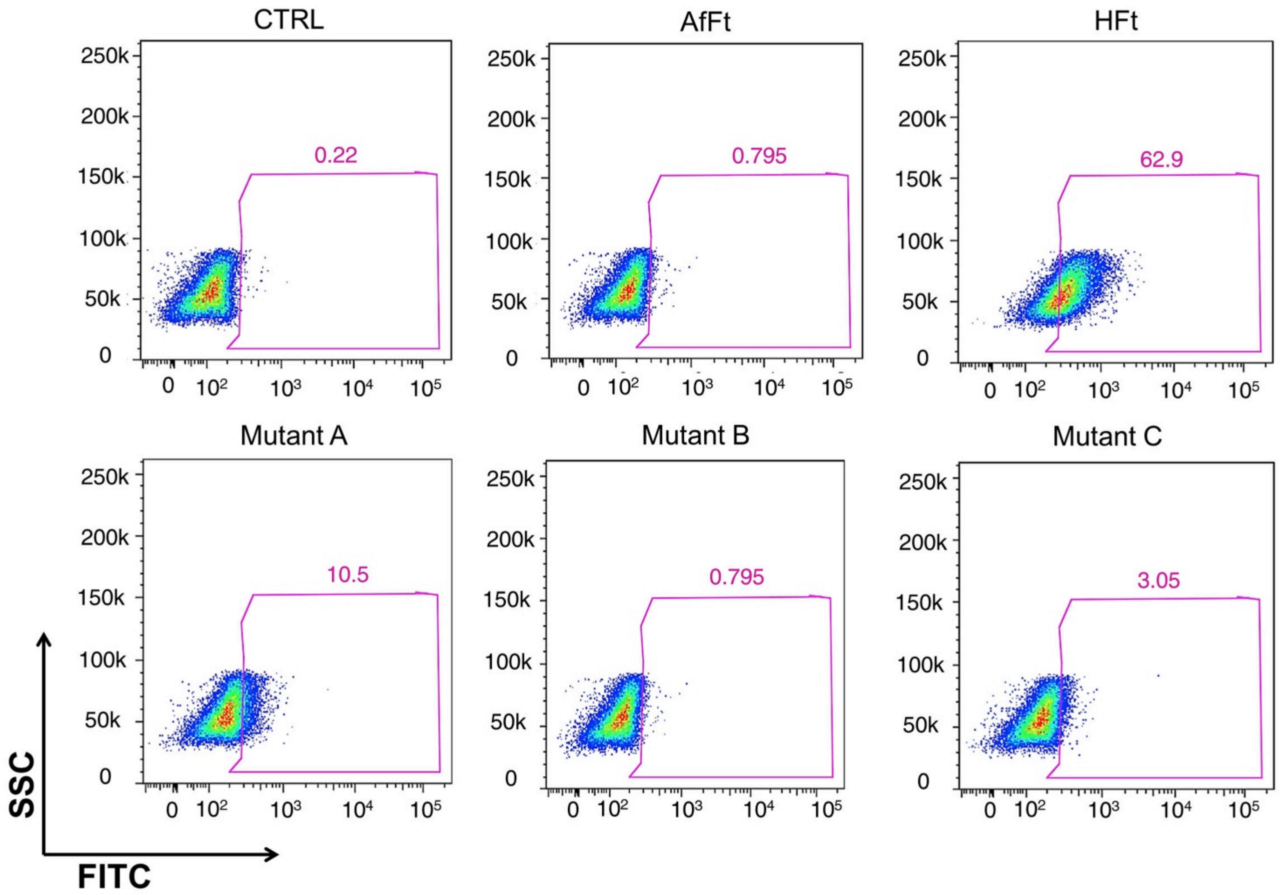

Supplementary Figure 5. Internalization analysis in HeLa cells by flow cytometry. Representative FACS plot from the data reported in Figure 3b, showing the uptake of FITC-conjugated AfFt, H-Ft, Mutant A, Mutant B, Mutant C in HeLa cells after 2 hours and 30 minutes. The gate for the specific signal was set based on the control sample (CTRL) and the percentage of FITC positive cells is indicated in pink in each plot. For each sample, 30,000 events were acquired. Source data are provided as a Source Data file.

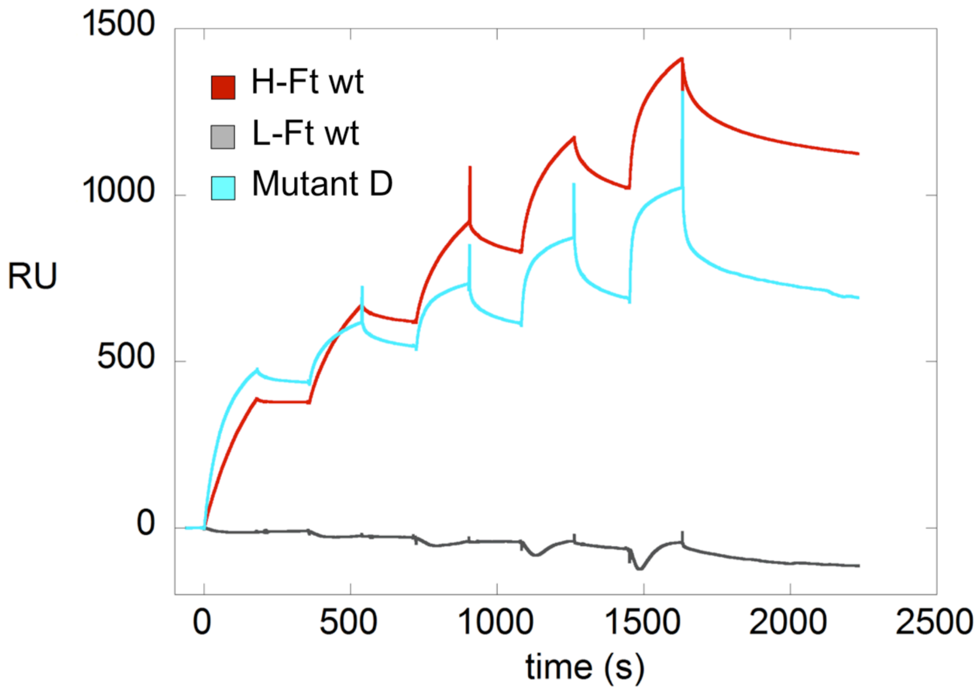

Supplementary Figure 6. Single kinetic SPR assay. SPR sensograms of the interaction between the immobilized his-tagged CD71 receptor and H-Ft wt (red line), L-Ft wt (grey line) and mutant D (cyan line), used as analytes. Analyte concentration is increased in five consecutive steps from 18.75 µg/mL to 300 µg/mL. Source data are provided as a Source Data file.

Supplementary Table 1. Cryo-EM data collection, refinement and validation statistics

	**CD71/H-Ft complex at 5.5 Å**<br>**EMD-0046**<br>**PDB 6GSR**	**CD71/H-Ft complex at 3.9 Å**<br>**EMD-0140**<br>**PDB 6H5I**
**Data collection and processing**		
Magnification	121K	107K
Voltage (kV)	300	300
Electron exposure (e-/Å^2)	40	40
Pixel size (Å)	1.33	1.15, 1.33
Symmetry imposed	C1	O, C1
Initial particle images (no.)	194501	43240
Final particle images (no.)	25870	53878
Map resolution (Å)	5.5	3.9
FSC threshold	0.143	0.143
Map resolution range (Å)	4.0-8.0	4.0-8.0
**Refinement**		
Initial model used (PDB code)	3KAS, 3AJO	3KAS, 3AJO
Model resolution (Å)	5.5	3.9
FSC threshold	0.143	0.143
Map sharpening *B* factor (Å^2)	-350	-272
Model composition		
Non-hydrogen atoms	45308	43900
Protein residues	5392	5392
B factors (Å^2)		
Proteins (min, max)	21.4 (5.5, 181.1)	169.2 (71.5, 505.5)
R.m.s. deviations		
Bond lengths (Å)	0.0059	0.0065
Bond angles (°)	1.00	1.11
Validation		
MolProbity score	1.45	1.36
Clashscore	5.94	2.7
Poor rotamers (%)	1.20	0.17
EMRinger score	1.06	1.92
Ramachandran plot		
Favored (%)	97.73	95.69
Allowed (%)	2.15	4.31
Disallawed (%)	0.11	0

Supplementary Table 2. Summary of interactions between CD71 and H-Ft

CD71	Group	Location	H-Ft	Group	Location	Distance (Å)	+exclusive/ common
			**Electrostatic interactions (hydrogen bonds, salt bridges)**				
Ser195	OG	loop βI-1-βII-1	Asp123	OD1	helix C	4.4	exclusive
Ser195	OG	loop βI-1-βII-1	Asp123	OD2	helix C	3.7	exclusive
Gln197	OE1	loop βI-1-βII-1	Lys119	NZ	helix C	3.3	exclusive
Gln197	NE2	loop βI-1-βII-1	Glu116	OE2	helix C	4.8	exclusive
Ser199	OG	strand βII-1	Asp15	OD1	helix A	4.8	exclusive
Ser199	OG	strand βII-1	Asp15	OD2	helix A	5.0	exclusive
Arg208	NH1	loop βII-1-βII-2	Thr5	N	N-term	3.7	common
Arg208	NH2	loop βII-1-βII-2	Thr5	O	N-term	4.0	common
Arg208	NH2	loop βII-1-βII-2	Arg79	NH2	loop BC	3.6	common
Val210	O	strand βII-2	Asn21	ND2	helix A	3.0	common
Asn215	OD1	loop βII-2-βII-3	Arg22	NH1	helix A	3.3	common
Asn215	OD1	loop βII-2-βII-3	Arg22	NH2	helix A	3.3	common
Asn215	ND2	loop βII-2-βII-3	Glu116	OE1	helix C	2.7	common
Asn215	ND2	loop βII-2-βII-3	Glu116	OE2	helix C	4.0	common
Lys344	NZ	helix αII-2	Asn25	OD1	helix A	3.6	common
Gly347	O	helix αII-2	*Lys87	NZ	*loop BC	3.6	common
Asn348	OD1	helix αII-2	*Lys87	NZ	*loop BC	3.0	common
Asn348	ND2	helix αII-2	Gln83	OE1	loop BC	3.5	common
Lys374	NZ	strand βII-8	Gln14	OE1	helix A	3.4	exclusive
Lys374	NZ	strand βII-8	Asp15	OD1	helix A	5.0	exclusive
			**Hydrophobic interactions**				
Ile201	CD1	strand βII-1	Ala18	CB	helix A	4.2	common
Ile201	CG2	strand βII-1	Ala18	CB	helix A	4.5	common
Leu209	CD1	strand βII-2	Gln14	CG	helix A	4.5	common
Leu209	CD2	strand βII-2	Arg79	CG	loop BC	3.8	common
Val210	CG1	strand βII-2	Gln14	CB	helix A	4.8	common
Val210	CG2	strand βII-2	Glu17	CB	helix A	4.4	common
Val210	CG2	strand βII-2	Glu17	CG	helix A	4.4	common
Val210	CG2	strand βII-2	Ala18	CB	helix A	4.4	common
Tyr211	CD1	strand βII-2	Asn21	CB	helix A	3.7	common
Tyr211	CE1	strand βII-2	Gln83	CB	loop BC	4.7	common
Tyr211	CE2	strand βII-2	Gln83	CG	loop BC	3.6	common
Tyr211	CE2	strand βII-2	Gln83	CD	loop BC	4.4	common
Tyr211	CD2	strand βII-2	Gln83	CD	loop BC	4.5	common
Tyr211	CG	strand βII-2	Phe81	CD2	loop BC	4.9	common
Leu212	CB	strand βII-2	Ala18	CB	helix A	3.6	common
Leu212	CB	strand βII-2	Arg22	CB	helix A	4.2	common
Leu212	CD2	strand βII-2	Ala19	CB	helix A	4.3	common
Leu212	CG	strand βII-2	Arg22	CG	helix A	4.7	common

CD71 residues are colored according to the color code used in Figure 2 panel a (right side).

Supplementary Table 3. Summary of the interfacing residues at CD71 apical domain contacted by the human H-Ft, GP1 of Machupo virus [3] and *Pv*RBP2b of *P. vivax* [4].

Human CD71	Human H-Ft	GP1 (MACV)	*Pv*RBP2b (*P. vivax*)
S195	K119, D123		
Q197	E116		
S199	D15		
I201	A18	F226	
D204		D114	
R208	T5, R79		N527, E530, D531
L209	Q14, R79, F81	S97, F98	
V210	Q14, E17, A18, N21	R111, F226, Y228	
Y211	F81, Q83	R111, S113, I115, V117	S541
L212	A18, A19, R22	P223, F226	Y542
V213			Y604
E214			K600
N215	R22, E116	E171	D603
A293		Y122	
E294		M119, Y122, K169	K600
E343	K86	Y122	
K344	N25	M119, D123, K169	Y604
N348	Q83, *K87	D114, S116	
S370		D114	
K371		S116	Y538
K374	Q14, D15		

CD71 residues contacted by H-Ft are colored according to the color code used in Figure 2 panel a (right side).

Supplementary Table 4. Kinetic and thermodynamic parameters for SPR experiments: heterogeneous analyte fit.

Protein Analyte	k_{on1} ($M^{-1}s^{-1}$)	k_{off1} (s^{-1})	K_{D1} (nM)	k_{on2} ($M^{-1}s^{-1}$)	k_{off2} (s^{-1})	K_{D2} (μM)
**H-Ft**	$(4.78\pm0.07)*10^{5}$	$(3.40\pm0.06)*10^{-3}$	7.1±0.2	$(1.69\pm0.04)*10^{5}$	$(2.51\pm0.06)*10^{-2}$	0.156±0.007
**Mutant A**	$(1.1\pm0.2)*10^{6}$	$(6.5\pm0.9)*10^{-1}$	570±170	$(1.4\pm0.1)*10^{4}$	$(2.7\pm 0.1)*10^{-2}$	1.9±0.3
**Mutant B**	$(1.5\pm0.3)*10^{6}$	$(3.3\pm0.2)*10^{-1}$	220±80	$(1.7\pm0.2)*10^{2}$	$(2.76\pm0.04)*10^{-2}$	158±18
**Mutant C**	Not feasible					

Supplementary Table 5. Kinetic and thermodynamic parameters for SPR experiments: simple 1:1 binding mode.

Protein Analyte	k_{on1} ($M^{-1}s^{-1}$)	k_{off1} (s^{-1})	K_{D1} (nM)
**H-Ft**	$(3.74 \pm 0.08)*10^4$	$(6.72 \pm 0.08)*10^{-4}$	17.9±0.6
**Mutant A**	$(1.7 \pm 0.2)*10^5$	$(1.8 \pm 0.2)*10^{-3}$	140±50
**Mutant B**	$(6.6 \pm 0.7)*10^4$	$(8.9 \pm 0.8)*10^{-3}$	130±30
**Mutant C**	1200 ±100	$(1.55 \pm 0.03)*10^{-3}$	1300 ±100

Supplementary Table 6. DNA sequences for all synthetic genes and primers used in this study.

H-Ft<br>(nucleotide sequence)	ACGACCGCGTCCACCTCGCAGGTGCGCCAGAACTACCACCAGGACTCAGAGGCCGCCATCAAC<br>CGCCAGATCAACCTGGAGCTCTACGCCTCCTACGTTTACCTGTCCATGTCTTACTACTTTGAC<br>CGCGATGATGTGGCTTTGAAGAACTTTGCCAAATACTTTCTTCACCAATCTCATGAGGAGAGG<br>GAACATGCTGAGAAACTGATGAAGCTGCAGAACCAACGAGGTGGCCGAATCTTCCTTCAGGAT<br>ATCAAGAAACCAGACTGTGATGACTGGGAGAGCGGGCTGAATGCAATGGAGTGTGCATTACAT<br>TTGGAAAAAAATGTGAATCAGTCACTACTGGAACTGCACAAACTGGCCACTGACAAAAATGAC<br>CCCCATTTGTGTGACTTCATTGAGACACATTACCTGAATGAGCAGGTGAAAGCCATCAAAGAA<br>TTGGGTGACCACGTGACCAACTTGCGCAAGATGGGAGCGCCCGAATCTGGCTTGGCGGAATAT<br>CTCTTTGACAAGCACACCCTGGGAGACAGTGATAATGAAAGC
H-Ft Mutant A<br>(nucleotide sequence)	CTCTAGAAATAATTTTGTTTAACTTTAAGAAGGAGATATACATATGACCACCGCAAGCACCAG<br>CCAGGTTCGTCAGAATTATCATGCAGCAAGCGAAGCAGCAATTAATGCACAGATTAATTTGGA<br>ACTGTATGCCAGCTATGTGTATCTGAGCATGAGCTATTATTTCGATCGTGATGATGTTGCCCT<br>GAAAAACTTCGCAAAATACTTTCTGCATCAGAGCCATGAAGAACGTGAACATGCAGAAAAACT<br>GATGAAACTGCAGAATCAGCGTGGTGGTCGTATCTTTCTGCAGGATATCAAAAAACCGGATTG<br>TGATGATTGGGAAAGCGGTCTGAATGCAATGGAATGTGCACTGCATCTGGAAAAAAATGTTAA<br>TCAGAGCCTGCTGGAACTGCATAAACTGGCAACCGATAAAAATGATCCGCATCTGTGCGATTT<br>TATCGAAACCCATTATCTGAACGAACAGGTGAAAGCCATTAAAGAACTGGGTGATCATGTTAC<br>CAATCTGCGTAAAATGGGTGCACCGGAAAGTGGTCTGGCAGAATACCTGTTTGATAAACATAC<br>CCTGGGTGATAGCGATAACGAAAGCTAATAAGGATCCGGCTGCTAACAAAGCCCGAAAGGAAG<br>CTGAGTTGGCTGCT
H-Ft Mutant B<br>(nucleotide sequence)	CTCTAGAAATAATTTTGTTTAACTTTAAGAAGGAGATATACATATGACCACCGCAAGCACCAG<br>CCAGGTTCGTCAGAATTATCATCAGGATAGCGAAGCAGCAATTAACCGTCAGATTAATTTGGA<br>ACTGTATGCCAGCTATGTGTATCTGAGCATGAGCTATTATTTCGATCGTGATGATGTTGCCCT<br>GAAAAACTTCGCAAAATACTTTCTGCATCAGAGCCATGAAGAACGTGAACATGCAGAAAAACT<br>GATGAAACTGCAGAATCAGCGTGGTGGTCGTATTGCACTGGCAGATATCAAAAAACCGGATTG<br>TGATGATTGGGAAAGCGGTCTGAATGCAATGGAATGTGCACTGCATCTGGAAAAAAATGTTAA<br>TCAGAGCCTGCTGGAACTGCATAAACTGGCAACCGATAAAAATGATCCGCATCTGTGCGATTT<br>TATCGAAACCCATTATCTGAACGAACAGGTGAAAGCCATTAAAGAACTGGGTGATCATGTTAC<br>CAATCTGCGTAAAATGGGTGCACCGGAAAGTGGTCTGGCAGAATACCTGTTTGATAAACATAC<br>CCTGGGTGATAGCGATAACGAAAGCTAATAAGGATCCGGCTGCTAACAAAGCCCGAAAGGAAG<br>CTGAGTTGGCTGCT
H-Ft Mutant C<br>(primer forward)	GCGTGGTGGTCGTATCGCTCTGGCGGATATCAAAAAACCGGATTG
H-Ft Mutant C<br>(primer reverse)	CAATCCGGTTTTTTGATATCCGCCAGAGCGATACGACCACCACGC
L-Ft<br>(nucleotide sequence)	AGCAGCCAGATTCGTCAGAATTATTCAACAGATGTTGAAGCAGCAGTTAATAGCCTGGTTAAT<br>CTGTATCTGCAGGCAAGCTATACCTATCTGAGCCTGGGTTTCTATTTTGATCGTGATGATGTT<br>GCACTGGAAGGTGTGAGCCATTTTTTTCGTGAACTGGCCGAAGAAAAACGCGAAGGTTATGAA<br>CGTCTGCTGAAAATGCAGAATCAGCGTGGTGGTCGTGCACTGTTTCAGGATATCAAAAAACCT<br>GCCGAAGATGAATGGGGTAAAACACCGGATGCAATGAAAGCAGCAATGGCACTGGAAAAAAAA<br>CTGAATCAGGCACTGCTGGATCTGCATGCACTGGGTAGCGCACGTACCGATCCGCATCTGTGT<br>GATTTTCTGGAAACCCACTTTCTGGATGAAGAAGTGAAACTGATTAAAAAGATGGGCGATCAT<br>CTGACCAATCTGCATCGTTTAGGTGGTCCGGAAGCAGGTCTGGGTGAATACCTGTTTGAACGC<br>CTGACACTGAAACACGAT
L-Ft Mutant D<br>(nucleotide sequence)	CTCTAGAAATAATTTTGTTTAACTTTAAGAAGGAGATATACATATGAGCAGCCAGATTCGTCA<br>GAATTATTCACAGGATGTTGAAGCAGCAGTTAATCGTCTGGTTAATCTGTATCTGCAGGCAAG<br>CTATACCTATCTGAGCCTGGGTTTCTATTTTGATCGTGATGATGTTGCACTGGAAGGTGTGAG<br>CCATTTTTTTCGTGAACTGGCCGAAGAAAAACGCGAAGGTTATGAACGTCTGCTGAAAATGCA<br>GAATCAGCGTGGTGGTCGTGCATTTTTTCAGGATATCAAAAAACCTGCCGAAGATGAATGGGG<br>TAAAACACCGGATGCAATGAAAGCAGCAATGGCACTGGAAAAAAAACTGAATCAGGCACTGCT<br>GGATCTGCATGCACTGGGTAGCGCACGTACCGATCCGCATCTGTGTGATTTTCTGGAAACCCA<br>CTTTCTGGATGAAGAAGTGAAACTGATTAAAAAGATGGGCGATCATCTGACCAATCTGCATCG<br>TTTAGGTGGTCCGGAAGCAGGTCTGGGTGAATACCTGTTTGAACGCCTGACACTGAAACACGA<br>TTAATAAGGATCCGAATTCGAGCTCCGTCGACAAGCTTGCGGCCGCACTCGAG
AfFt<br>(nucleotide sequence)	GCAAGCATCAGCGAAAAAATGGTTGAAGCACTGAATCGTCAGATTAACGCAGAAATCTATAGC<br>GCCTATCTGTATCTGAGCATGGCGAGCTATTTTGATAGCATTGGTCTGAAAGGCTTTAGCAAT<br>TGGATGCGTGTTCAGTGGCAAGAGGAACTGTGTCATGCAATGAAAATGTTTGATTTTGTGAGC<br>GAACGCGGCGGCCGCGTGAAACTGTATGCGGTGGAAGAACCGCCGAGCGAATGGGATAGTCCG<br>CTGGCTGCTTTTGAACATGTTTATGAACATGAAGTGAACGTGACCAAACGCATTCATGAACTG<br>GTTGAAATGGCCATGCAAGAAAAAGATTTTGCGACCTATAACTTCCTGCAGTGGTATGTTGCA<br>GAACAGGTTGAAGAAGAAGCGAGCGCACTGGATATTGTTGAAAAACTGCGTCTGATTGGTGAA<br>GATAAACGTGCACTGCTGTTTCTGGATAAAGAACTGAGCCTGCGCCAGTTTACCCCTCCGGCA<br>GAAGAAGAGAAA

Supplementary References

1 Larkin, M. A. *et al.* Clustal W and Clustal X version 2.0. *Bioinformatics* **23(21)**, 2947-2948 (2007).

2 Niitsu, Y. & Listowsky, I. Mechanisms for the formation of ferritin oligomers. *Biochemistry* **12(23)**, 4690-4695 (1973).

3 Radoshitzky, S. R. *et al.* Machupo virus glycoprotein determinants for human transferrin receptor 1 binding and cell entry. *Plos One* **6**, e21398 (2011).

4 Gruszczyk, J. *et al.* Cryo-EM structure of an essential Plasmodium vivax invasion complex. *Nature* **559(7712)**, 135-139 (2018).
