# Supplementary material for: Cryo-EM structure of the human ferritin–transferrin receptor 1 complex
Source: Nat Commun. 2019 Mar 8;10:1121. doi: 10.1038/s41467-019-09098-w (PMC6408514; doi:10.1038/s41467-019-09098-w)
Supplement: Supplementary file 3 — Reporting Summary Checklist [file 41467_2019_9098_MOESM3_ESM.pdf]

nature research

Corresponding author(s): Amedee des Georges, Beatrice Vallone

☐ Initial submission ☐ Revised version ☒ Final submission

Reporting Summary

Nature Research wishes to improve the reproducibility of the work that we publish. This form provides structure for consistency and transparency in reporting. For further information on Nature Research policies, see Authors & Referees and the Editorial Policy Checklist.

Please do not complete any field with "not applicable" or n/a. Refer to the help text for what text to use if an item is not relevant to your study.
For final submission: please carefully check your responses for accuracy; you will not be able to make changes later.

Statistical parameters

When statistical analyses are reported, confirm that the following items are present in the relevant location (e.g. figure legend, table legend, main text, or Methods section).

n/a	Confirmed	
☐	☒	The exact sample size (*n*) for each experimental group/condition, given as a discrete number and unit of measurement
☐	☒	An indication of whether measurements were taken from distinct samples or whether the same sample was measured repeatedly
☒	☐	The statistical test(s) used AND whether they are one- or two-sided<br>*Only common tests should be described solely by name; describe more complex techniques in the Methods section.*
☒	☐	A description of all covariates tested
☒	☐	A description of any assumptions or corrections, such as tests of normality and adjustment for multiple comparisons
☐	☒	A full description of the statistics including central tendency (e.g. means) or other basic estimates (e.g. regression coefficient) AND variation (e.g. standard deviation) or associated estimates of uncertainty (e.g. confidence intervals)
☒	☐	For null hypothesis testing, the test statistic (e.g. *F*, *t*, *r*) with confidence intervals, effect sizes, degrees of freedom and *P* value noted<br>*Give P values as exact values whenever suitable.*
☒	☐	For Bayesian analysis, information on the choice of priors and Markov chain Monte Carlo settings
☒	☐	For hierarchical and complex designs, identification of the appropriate level for tests and full reporting of outcomes
☒	☐	Estimates of effect sizes (e.g. Cohen's *d*, Pearson's *r*), indicating how they were calculated
☐	☒	Clearly defined error bars<br>*State explicitly what error bars represent (e.g. SD, SE, CI)*

Our web collection on statistics for biologists may be useful.

Software and code

Policy information about availability of computer code

Data collection	Cryo-EM data collection: Leginon and EPU
Data analysis	Cryo-EM data processing: MotionCor2, Gctf v 1.06, RELION 2.0, cisTEM<br>Cryo-EM map resolution: ResMap implemented in RELION 2.0<br>Cryo-EM model building and refinement: Phenix 1.13-2998, Chimera 1.12 and COOT 0.7<br>Cryo-EM model validation: MolProibity and EMRinger implemented in Phenix 1.13-2998 package<br>Structure Visualization:Chimera 1.12 and MacPymol 1.0<br>SPR data analysis: Biacore X100 evaluation software<br>Data Plotting: Matlab 2018<br>Flow cytometry: FlowJo 9.3.4 software

For manuscripts utilizing custom algorithms or software that are central to the research but not yet described in published literature, software must be made available to editors/reviewers upon request. We strongly encourage code deposition in a community repository (e.g. GitHub). See the Nature Research guidelines for submitting code & software for further information.

April 2018

Data

Policy information about availability of data

All manuscripts must include a data availability statement. This statement should provide the following information, where applicable:

- Accession codes, unique identifiers, or web links for publicly available datasets
- A list of figures that have associated raw data
- A description of any restrictions on data availability

All data supporting the findings of this study are available within this paper. The cryo-EM maps of CD71/H-Ft complex at 5.5 Å and at 3.9 Å and coordinates generated and analysed in the current study have been deposited in the Electron Microscopy Data Bank and in the Protein Data Bank under accession code EMD-0046 (PDB 6GSR) and EMD-0140 (PDB 6H5I), respectively.

Field-specific reporting

Please select the best fit for your research. If you are not sure, read the appropriate sections before making your selection.

☒ Life sciences ☐ Behavioural & social sciences ☐ Ecological, evolutionary & environmental sciences

Life sciences study design

All studies must disclose on these points even when the disclosure is negative.

Sample size	HeLa cell apo-ferritin uptake was performed by flow cytometry (FACS) to observe the capability of endogenous CD71 receptor to internalize ferritin mutants in comparison to the wild type and an archeal ferritin used as a positive and negative controls, respectively. Previous analyses conducted in our laboratory and present in literature show that three independent and internally consistent replicates per sample would provide a reliable statistics to assess and quantify ferritin uptake occurrence or abolishment.
Data exclusions	No data was excluded from this study.
Replication	HeLa cell internalization assay consists of three independent biological replicates. FACS data were acquired on 30,000 events.<br>The uptake experiment was further assessed at the confocal microscope, showing consistency with FACS data.
Randomization	HeLa cells were randomly allocated to each internalization condition.
Blinding	Investigators were not blinded during data collection since data for internalization included negative and positive controls and were quantitatively assessed by FACS experiments.

Reporting for specific materials, systems and methods

We require information from authors about some types of materials, experimental systems and methods used in many studies. Here, indicate whether each material, system or method listed is relevant to your study. If you are not sure if a list item applies to your research, read the appropriate section before selecting a response.

Materials & experimental systems

n/a	Involved in the study	
☒	☐	Unique biological materials
☒	☐	Antibodies
☐	☒	Eukaryotic cell lines
☒	☐	Palaeontology
☒	☐	Animals and other organisms
☒	☐	Human research participants

Methods

n/a	Involved in the study	
☒	☐	ChIP-seq
☐	☒	Flow cytometry
☒	☐	MRI-based neuroimaging

Eukaryotic cell lines

Policy information about cell lines

Cell line source(s)	HeLa cells (ATCC Number: CCL-2)
Authentication	None of the cell lines used were authenticated.
Mycoplasma contamination	Cell lines were not tested for mycoplasma contamination but clear cells were observed under the microscope.

Commonly misidentified lines (See ICLAC register)	No misidentified cell lines were used.

Flow Cytometry

Plots

Confirm that:

☒ The axis labels state the marker and fluorochrome used (e.g. CD4-FITC).

☒ The axis scales are clearly visible. Include numbers along axes only for bottom left plot of group (a 'group' is an analysis of identical markers).

☒ All plots are contour plots with outliers or pseudocolor plots.

☒ A numerical value for number of cells or percentage (with statistics) is provided.

Methodology

Sample preparation	HeLa cell line used for experiments was available in our laboratory. Cells were used after thawing and after two days in medium culture for recovering.
Instrument	FACS data were acquired using a BD LSRFortessa (BD Biosciences, San Jose, CA, USA). The confocal laser-scanning microscope used was an Olympus FV10i platform.
Software	FACS data were acquired using the FACSDiva software (BD Biosciences version 6.1.3) and analyzed with FlowJo 9.3.4 software.
Cell population abundance	Flow cytometry analysis and microscope observations showed healthy and homogeneous cell population.
Gating strategy	The gate for FITC signal was set using the untreated sample as reference for zero signal and the percentage of FITC positive cells was calculated for each condition, as exemplified in Extended data Figure 5.

☐ Tick this box to confirm that a figure exemplifying the gating strategy is provided in the Supplementary Information.

This checklist template is licensed under a Creative Commons Attribution 4.0 International License, which permits use, sharing, adaptation, distribution and reproduction in any medium or format, as long as you give appropriate credit to the original author(s) and the source, provide a link to the Creative Commons license, and indicate if changes were made. The images or other third party material in this article are included in the article's Creative Commons license, unless indicated otherwise in a credit line to the material. If material is not included in the article's Creative Commons license and your intended use is not permitted by statutory regulation or exceeds the permitted use, you will need to obtain permission directly from the copyright holder. To view a copy of this license, visit http://creativecommons.org/licenses/by/4.0/
